# Microbiota-Derived Extracellular Vesicles Promote Immunity and Intestinal Maturation in Suckling Rats

**DOI:** 10.3390/nu15214701

**Published:** 2023-11-06

**Authors:** Sergio Martínez-Ruiz, Laura Sáez-Fuertes, Sergi Casanova-Crespo, María J. Rodríguez-Lagunas, Francisco J. Pérez-Cano, Josefa Badia, Laura Baldoma

**Affiliations:** 1 Departament de Bioquímica i Fisiologia, Facultat de Farmàcia i Ciències de l’Alimentació, Universitat de Barcelona, 08028 Barcelona, Spain; sergio_martinez_ruiz@ub.edu (S.M.-R.); laurasaezfuertes@ub.edu (L.S.-F.); sergi.casanova@ub.edu (S.C.-C.); josefabadia@ub.edu (J.B.); 2 Institut de Biomedicina de la Universitat de Barcelona (IBUB), 08028 Barcelona, Spain; 3 Institut de Recerca Sant Joan de Déu (IRSJD), 08950 Barcelona, Spain; 4 Nutrition and Food Safety Research Institute (INSA-UB), 08921 Santa Coloma de Gramenet, Spain

**Keywords:** early life, extracellular vesicles, microbiota–host crosstalk, probiotic, *E. coli* Nissle 1917, immunity, intestinal permeability

## Abstract

Microbiota–host communication is primarily achieved by secreted factors that can penetrate the mucosal surface, such as extracellular membrane vesicles (EVs). The EVs released by the gut microbiota have been extensively studied in cellular and experimental models of human diseases. However, little is known about their in vivo effects in early life, specifically regarding immune and intestinal maturation. This study aimed to investigate the effects of daily administration of EVs from probiotic and commensal *E. coli* strains in healthy suckling rats during the first 16 days of life. On days 8 and 16, we assessed various intestinal and systemic variables in relation to animal growth, humoral and cellular immunity, epithelial barrier maturation, and intestinal architecture. On day 16, animals given probiotic/microbiota EVs exhibited higher levels of plasma IgG, IgA, and IgM and a greater proportion of Tc, NK, and NKT cells in the spleen. In the small intestine, EVs increased the villi area and modulated the expression of genes related to immune function, inflammation, and intestinal permeability, shifting towards an anti-inflammatory and barrier protective profile from day 8. In conclusion, interventions involving probiotic/microbiota EVs may represent a safe postbiotic strategy to stimulate immunity and intestinal maturation in early life.

## 1. Introduction

The intestinal microbiota plays a key role in the development and function of the intestinal immune system, reinforces the intestinal epithelial barrier, and contributes to host defense responses against pathogens [1]. The gut microbiota is considered an essential organ that establishes multidirectional communication with other host organs through several routes including endocrine, immunological, metabolic, and neural pathways [2]. Thus, alterations in the gut microbiota composition and diversity not only cause gut-related disorders but also contribute to extraintestinal diseases [3,4]. 

The gut barrier is essential for maintaining the segregation between the luminal microbial community and the host [5,6]. The barrier function is accomplished by a combination of several mechanisms, which include the mucus layer, host-secreted immunoglobulin (Ig) A and antimicrobial peptides, the intestinal epithelium, and the mucosal immune system. The intestinal epithelium is formed by a single layer of polarized cells sealed by tight junctions (TJs) that restrict the paracellular passage of large molecules [7,8]. In adulthood, the passage of molecules through the intestinal barrier is selectively controlled by specific endocytic pathways and regulation of TJs. In contrast, the intestine is highly permeable during early life, especially in rodents. This is due to the characteristics of immature intestinal epithelial cells, which have high endocytic capacity (transcytosis) and altered expression of TJ proteins that leads to enhanced paracellular passage. Reduced expression of strengthening TJ proteins (i.e., occludin, ZO-1) and overexpression of pore-forming proteins such as claudin-2 have been observed in the intestinal tissue of infant mice [9]. In the neonatal period, increased intestinal permeability contributes to the maturation of the immune system by transferring microbial components and antigens to the underlying immune cells [10]. Moreover, intestinal transfer of maternal IgG antibodies through endocytosis mediated by the neonatal Fc receptor for IgG (FcRn) provides passive immune protection to the offspring [11]. The leaky barrier properties gradually disappear as functional intestinal maturation progresses. The timing and extent of the enhanced endocytic and paracellular transfer activity before gut closure depend on the animal species [10].

The structure of the mature intestinal mucosal barrier prevents direct contact of the gut bacteria with intestinal epithelial and immune cells. Therefore, communication between commensal microbiota and host cells is mainly achieved by secreted bacterial components that can penetrate the mucosal surface. In addition to secreted proteins, metabolites, and signaling molecules [12,13,14], the gut microbiota releases extracellular vesicles (EVs). These EVs can cross the mucus layer, pass through the epithelial monolayer, and disseminate in the body to perform their actions [15,16]. There is growing evidence that interkingdom crosstalk in the gut is mainly mediated by EVs [17].

Bacterial EVs are nano-size spherical structures (20–300 nm) derived from bacterial membranes. They serve as a secretion system for molecules that are essential for bacterial survival and interspecies communication. During the biogenesis process, EVs selectively enclose a wide variety of bacterial molecules that include characteristic microbe-associated molecular patterns (MAMPs), proteins, metabolites, DNA, and regulatory RNAs. The vesicle cargo depends on the producer bacterial strain and its physiological state. This fact determines functions that are strain specific [18]. In the context of the gut microbiota, cumulative evidence shows that bacterial EVs mediate microbiota functions by transferring effector molecules into host cells (reviewed in [17]). MAMPs on the vesicle surface such as lipopolysaccharide (LPS) interact with extracellular immune receptors, known as pattern-recognition receptors (PRRs) of the host cells to activate signaling cascades to control immune and defense responses [19,20]. Moreover, EVs are taken up by epithelial and immune cells through endocytic pathways [21]. This process allows for the intracellular delivery of MAMPs and other active bacterial molecules that trigger specific host responses. In this scenario, continuous activation of immune receptors by EVs released by gut microbiota results in controlled basal inflammation that is essential to maintain balanced immune responses to preserve intestinal homeostasis [22]. Thus, we hypothesized that in early life, when the intestine is more permeable, the internalization rate of microbiota EVs could increase, and therefore, it could have a greater impact on the developing host.

The study of gut microbiota EVs and their actions on host physiology is an emerging research area. Many reports on EVs from probiotics (*Lactobacillus* sp., *Bifidobacterium* sp.) and gut beneficial bacteria (*Bacteroides fragilis*, *Bacteroides thetaiotaomicron*, *Akkermansia muciniphila*, *Escherichia coli*) prove their immunomodulatory and barrier-strengthening effects in both in vitro and in vivo models through different mechanisms. Depending on the producer bacterial strain, EVs can regulate the expression of TJ proteins, antimicrobial peptides, and mucus production and induce innate and/or adaptative responses [17,23,24]. In this context, our group has contributed to this knowledge using *E. coli* strains as a model of microbiota-derived vesicles, specifically the probiotic *E. coli* Nissle 1917 (EcN) and commensal *E. coli* strains isolated from the stools of healthy human individuals. We have shown that EVs from these strains activate immune responses and modulate the barrier integrity in intestinal epithelial cells and colonic explants [22,25]. Moreover, EVs from these microbiota strains modulate human dendritic cells and the subsequent T-helper (Th) responses through mechanisms that are strain specific [26].

While microbiota EVs have been extensively studied in cellular and experimental models of human diseases such as inflammatory bowel disease, obesity, diabetes, or neurological disorders [27,28,29,30,31,32,33], little is known about their in vivo effects in early life, specifically regarding immune and intestinal maturation during the neonatal period. The present study aimed to investigate the effects of daily administration of EVs from probiotic and commensal *E. coli* strains in healthy suckling rats during the first 16 days of life. We evaluated intestinal and systemic variables in relation to animal growth, humoral and cellular immunity, epithelial barrier maturation and function, and intestinal architecture.

## 2. Materials and Methods

### 2.1. Bacterial Strains and Isolation of EVs

The probiotic *E. coli* Nissle 1917 (EcN) was provided by Ardeypharm (GmbH, Herdecke, Germany). The *E. coli* strain EcoR12, included in the ECOR reference collection, was isolated from a healthy human stool sample [34].

Bacterial EVs were obtained as described previously [26]. Briefly, EcN and EcoR12 strains were grown in Luria–Bertani broth (LB) for 16 h. Bacterial cells were pelleted by centrifugation and the culture supernatants were filtered through 0.22 µm filters (Merck, Millipore, MA, USA) to remove residual bacteria. The filtrated supernatants were concentrated using centrifugal Centricon Plus-70 100 kDa filters (Millipore). After an additional filtration step using 0.22 μm pore size filters, the vesicles were collected by ultracentrifugation at 150,000× *g* for 1 h at 4 °C. The resulting pellets were washed twice in phosphate-buffered saline (PBS). Finally, pelleted EVs were resuspended in PBS, aliquoted, and stored at −20 °C until use. Sterility of the samples was confirmed on LB plates. Quantification of samples was assessed in terms of the protein and lipid content of EVs using, respectively, the Pierce BCA protein assay and the lipophilic fluorescent dye FM4-64 (Thermo Fisher Scientific, Barcelona, Spain), which intercalates into the vesicle membrane. For the FM4-64 assay, EV samples were incubated with FM4-64 (5 μg/mL in PBS) for 10 min at room temperature. Reactions containing only EVs or the FM4-64 probe were used as negative controls. After excitation at 515 nm, emission at 640 nm was measured with the Varioskan Lux multiplate reader (Thermo Fisher Scientific) [35]. Fluorescence was normalized by protein concentration.

### 2.2. Animals

A total of nine pregnant Lewis rats (G14), purchased from Janvier (Le Genest-St-Isle, France), were individually housed in cages (2184 L Eurostandard Type II L, Tecniplast, West Chester, PA, USA). All cages contained tissue paper and fibrous particle bedding. Rats were monitored daily until natural delivery. Litters were randomly distributed into three experimental groups (with 3 lactating dams each) and the number of pups was unified to 8 per dam, keeping a similar proportion (40–60%) of males and females per litter. The rats were housed in controlled conditions of temperature, humidity, and light: dark cycles of 12 h, in an isolated room, designed and authorized for working under biosecurity level 2 conditions, at the Animal Experimentation Unit (UEA) of the Diagonal Campus at the University of Barcelona (UB). Rats were fed with a commercial diet corresponding to the American Institute of Nutrition (AIN) 93 G formulation (Teklad Global Diet 2014, INOTIV, Indianapolis, IN, USA) [36] and water ad libitum. Neonatal rats had free access to maternal milk. For animal handling, the mother was placed in a new cage and the pups were kept in the home cage. Oral administration to each experimental group was performed randomly, and then the dams were brought together with the corresponding litters. Animal procedures were approved by the Ethical Committees for Animal Experimentation (CEEA) of the UB and the Catalonian Government (CEEA-UB Ref.169/20 and Ref.11461, respectively) in compliance with the EU Directive 2010/63/EU, which establishes the rules for the protection of animals used in experimental work.

### 2.3. Experimental Design

Litters were randomly distributed into three experimental groups: the control group, and animals receiving EVs from the probiotic EcN (EcN EVs) or EVs from the commensal EcoR12 (EcoR12 EVs). Each experimental group was formed by 3 litters of 8 pups each (*n* = 24/group). Neonatal rats were orally administered from the second (day 2) to the sixteenth day of life (day 16). Since the pups gained weight through the experimental period, during the first eight days, the EcN EVs and EcoR12 EVs groups were given a concentration of EVs of 2 µg protein/animal/day (body weight between 5.5 and 12 g). From the ninth day of life, the administered dose was increased to 5 µg protein/animal/day until the last day of the procedure (body weight from 12 to 29 g). The administered volume was adjusted to 100 µL. The control group received a matched volume of the vehicle (PBS). The selected dose of EVs was based on previous studies in mice with an average body weight of 20–22 g [33]. To confirm that equal amounts of EcN EVs and EcoR12 EVs were administered, the stock EV samples (40 µg/mL) were diluted and quantified with the lipophilic fluorescent dye FM4-64. Fluorescence intensity values did not differ between samples (at 8 µg/mL protein concentration the fluorescence values were 0.96 ± 0.025 for EcN EVs and 0.94 ± 0.03 for EcoR12 EVs). Oral administration was performed as described previously [33,37], using low-capacity syringes (Hamilton Bonaduz, Bonaduz, Switzerland). Half of the animals of each litter were euthanized on day 8 and the other half on day 16. For this purpose, animals were randomly selected, maintaining similar proportions of males and females at both intervention times.

Body weight was measured daily. To prevent adverse intestinal effects of EcN or EcoR12 EVs in neonatal rats, fecal samples were collected during the first two weeks of life (days 4–7 and days 8–11) by gentle abdominal massage. Fecal specimens were weighed and scored in a blinded manner (from 1–4) based on their color, texture, and amount. The fecal score (FS) values were: (1) normal feces, (2) soft yellow-green feces, (3) totally loose yellow-green feces, and (4) high amount of watery feces, as in previous studies [37].

### 2.4. Sample Collection

At the end of the experimental procedure (day 8 or day 16 of life) neonatal rats were anesthetized with intramuscular injection of ketamine/xylazine (Imalgene 100 mg/mL, Merial Laboratorios, Barcelona, Spain/Rompun® 20 mg/mL, Bayer Hispania, Sant Joan Despí, Spain). At this point, the naso-anal and tail lengths were measured to obtain the body/tail ratio. The body mass index (BMI) was calculated as body weight/length^2^ (g/cm^2^) and the Lee index as 3√weight/length × 1000 (3√g/cm). Once the animals were fully unconscious, euthanasia was performed by exsanguination. Blood was collected by cardiac puncture to obtain plasma samples, which were stored at −20 °C. The spleen, thymus, liver, stomach, small intestine, cecum, kidneys, and heart were obtained and weighed to obtain morphogenic variables. Also, the length of the small intestine was measured. A central portion (1 cm) of the small intestine was placed in liquid nitrogen directly and then stored at −80 °C for gene expression analysis. A fragment of the distal jejunum was collected and processed for histological analysis. The gut wash was obtained from the remaining parts of the intestine as described previously [37]. The spleens were processed for lymphocyte isolation as described below.

### 2.5. Quantification of Immunoglobulins

Plasma concentrations of IgG isotypes (IgG1, IgG2a, IgG2b, IgG2c), IgA, and IgM were quantified using ProcartaPlex™ Multiplex immunoassay (Thermo Fisher Scientific, Barcelona, Spain), as previously described [37]. Briefly, specific color-coded capture beads were bound to the Ig of interest. After adding the different detection antibodies conjugated to phycoerythrin (PE), the concentration of each analyte was obtained using the MAGPIX® analyzer (Luminex Corporation, Austin, TX, USA) at the Cytometry Service of the Scientific and Technological Centers of the University of Barcelona (CCiT-UB).

### 2.6. Gene Expression Analysis by Reverse Transcription Quantitative PCR (RT-qPCR)

Small intestine tissue collected as described above (Section 2.4) was homogenized in lysing matrix tubes (MP Biomedicals, Illrich, France) using Fast-Prep-24 equipment (MP Biomedicals) [37]. RNA was isolated with the RNeasy® Mini Kit (Qiagen, Madrid, Spain). RNA purity and concentration were assessed using a NanoPhotometer (BioNova Scientific S.L., Fremont, CA, USA). Then, cDNA was synthesized using the TaqMan® Reverse Transcription Reagents (Applied Biosystems, AB, Weiterstadt, Germany) following the manufacturer’s protocol.

Quantitative PCR was carried out using the ABI Prism 7900 HT equipment. The specific PCR TaqMan® primers (AB) were: *TLR2* (Rn02133647_s1), *TLR7* (Rn01771083_s1), *IGA* (Rn01511082_m1), *CD68* (Rn00566655_m1), *FcRn* (Rn00583712_m1), *MUC2* (Rn01498206_m1), *IL12* (Rn00584538_m1), *COX-2* (Rn01483828_m1), *OCLN* (Rn00580064_m1), and *ZO-1* (Rn02116071_s1). The relative gene expression was normalized with the endogenous housekeeping gene *Gusb* (Rn00566655_m1) by means of the 2-ΔΔCt method. The results were expressed as the percentage of expression in each experimental group, normalized to the mean value obtained for the control group, which was set at 100%.

### 2.7. Lymphocyte Isolation from Spleen

Spleens were passed through a sterile mesh cell strainer (40 μm, Thermo Fisher Scientific, S.L.U, Barcelona, Spain) [38]. Then, erythrocytes were removed by osmotic lysis and lymphocytes were suspended in Roswell Park Memorial Institute medium (RPMI) supplemented with 10% heat-inactivated fetal bovine serum (FBS), 100 IU/mL streptomycin–penicillin, 2 mM L-glutamine. Dead cells were stained with Trypan Blue. Cell numbers and viability were measured using a Countess^TM^ Automated Cell Counter (Invitrogen^TM^, Thermo Fisher Scientific, S.L.U, Barcelona, Spain).

### 2.8. Lymphocyte Phenotypic Analysis by Immunofluoroscence Staining and Flow Cytometry

The phenotype of spleen lymphocytes was analyzed by using fluorescent monoclonal antibodies (Ab), as previously described [38]. The Ab used were conjugated to the fluorochromes fluorescein isothiocyanate (FITC), phycoerythrin (PE), peridinin chlorophyll protein (PerCP), allophycocyanin (APC), brilliant violet 421 (BV421), brilliant violet 605 (BV605), or brilliant violet 786 (BV786). Ab used in this study were specific against rat and fluorochrome conjugated as follows: FITC-TCRαβ, Bv786-CD8β, FITC-CD25, PE-CD161a, Bv605-TCRγδ, PE-CD4, PerCP-CD8α, APC-CD4, and BV421-CD45RA (BD Biosciences) and APC-FoxP3 (eBioscience, Frankfurt, Germany). Briefly, lymphocytes were incubated with saturating concentrations of fluorochrome-conjugated Ab, fixed with 0.5% p-formaldehyde and stored (4 °C, in darkness) until flow cytometry analysis. For Treg analysis, lymphocytes were incubated (20 min, 4 °C, in darkness) with fluorochrome-conjugated anti-CD4 (PE) and anti-CD25 (FITC) Ab, treated (30 min, 4 °C, in darkness) with a fixation–permeabilization solution (eBioscience) and then incubated (30 min, 4 °C, in darkness) with APC-conjugated anti-Foxp3 Ab (eBioscience). A negative control staining with an isotype-matched monoclonal Ab was included in each cell sample.

Analyses were performed with an AURORA^TM^ Cytometer (Beckman Coulter, Miami, FL, United States) in the Flow Cytometry Unit of the Scientific and Technological Centers of the UB (CCiT-UB), and data were analyzed by Flowjo v10 software (Tree Star, Inc., Ashland, OR, USA). The results were expressed as percentages of positive cells in the lymphocyte population selected according to their forward-scatter characteristics (*FSCs*) and side-scatter characteristics (*SSCs*) or in a particular lymphocyte population.

### 2.9. Histomorphometry Analysis

Distal jejunum fragments obtained from neonatal rats were processed for hematoxylin–eosin staining as previously described [37]. Intestinal morphology was analyzed by bright-field microscopy (Olympus BX41, Olympus Corporation, Shinjuku, Tokyo, Japan) at 100x magnification. The morphometric measurements were performed with ImageJ v1.53t. Ten villi from each animal were randomly selected to measure their height, width, and area. The villi width was assessed at the crypt–villus junction. The villi area was calculated by delineating the region of interest (excluding the crypts). Crypt depths were also determined. Statistical analysis was carried out with the mean value of the parameters calculated for each animal.

### 2.10. Statistical Analysis

For statistical analysis, the Statistical Package for Social Sciences (SPSS v22.0) (IBM, Chicago, IL, USA) was used. Data were tested for normal distribution (by Shapiro–Wilk) and variance equality (by Levene’s test). A conventional one-way ANOVA test was carried out followed by the post hoc Bonferroni test when data were homogeneous and had a normal distribution, considering the experimental group as the independent variable. The non-parametric Kruskal–Wallis test followed by the post hoc Dunn’s multiple comparison test was performed when data displayed non-normal distribution or dissimilar variances and for non-parametric variables such as the feces scoring. Differences were considered significant at *p* < 0.05. The results were expressed as mean ± SEM (*n* animals).

## 3. Results

### 3.1. Growth and Morphometry Variables

**Figure 1:**
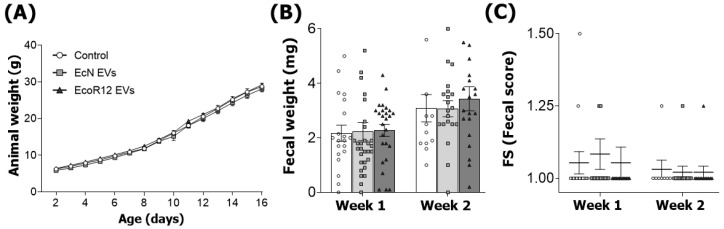
Interventions with EcN EVs or EcoR12 EVs do not affect body weight and feces features. The experimental groups were: Control (white circles), EcN EVs (gray squares), and EcoR12 EVs (black triangles). (**A**) Body weight (g) for control, EcN EVs, and EcoR12 EVs groups was measured daily over the 16-day experimental period. (**B**) The weight (mg) and (**C**) consistency of stool specimens were recorded during the first two weeks for the three experimental groups. Stool consistency was scored as described in the Materials and Methods to obtain the FS. Results are expressed as mean ± SEM (*n* = 12–24 animals/group).

The body weight was monitored daily until the last day of the intervention (Figure 1A). Pups receiving EcN or EcoR12 EVs have similar weights compared to the control animals. Concerning the impact of the intervention on stool features, administration of EcN or EcoR12 EVs during the first two weeks of life had no significant impact on either fecal weight or the stool consistency. In this sense, the FS value (scale 1–4) was close to 1 for all groups (Figure 1B,C).

In addition, other growth-related parameters and the relative weight of organs were measured on day 8 and day 16 of life, without great changes due to EcN or EcoR12 EVs interventions (Table 1).

On day 8, only minor differences (*p* < 0.05) were observed in the EcoR12 EVs interventional group affecting the body mass, which were no longer evident on day 16. Concerning the relative organ weight, some small differences were observed between the two interventions. In this sense, the spleen weight was higher in animals given EcN EVs, both on day 8 and day 16. Moreover, oral administration of EcN EVs also resulted in a small significant increase in heart weight only on day 8. However, by day 16, the relative weight of this organ in the EcN EVs group was comparable to that of the control animals. In contrast, the intervention with EcoR12 EVs only affected the empty cecum relative weight, which had a slightly higher increase on day 8 (0.05% vs. control) and day 16 (0.04% vs. control).

The hematological variables were also assessed in blood samples at the two final time points (days 8 and 16) (Supplementary Table S1). No significant differences were apparent between the three experimental groups.

### 3.2. EVs from EcN and EcoR12 Stimulate Immunoglobulin Production in Neonatal Rats

**Figure 2:**
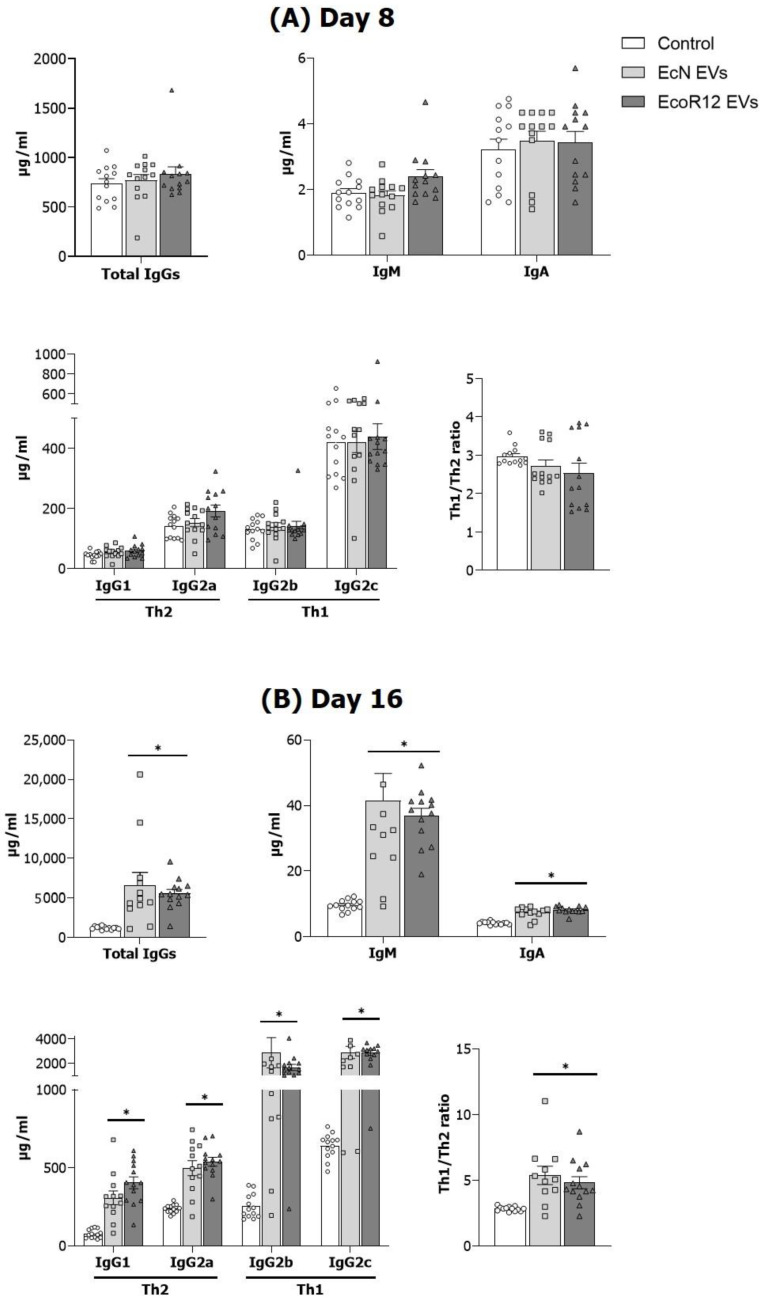
Effect of the interventions with EcN and EcoR12 EVs on the Ig levels in plasma on days 8 (**A**) and 16 (**B**). The three experimental groups were: Control (white bars), EcN EVs (light gray bars), and EcoR12 EVs (dark gray bars). The Th1/Th2 Ig ratio applies to the relation (IgG2b + IgG2)/(IgG1 + IgG2a). Results are expressed as ± SEM (*n* = 12 animals/group at both days 8 and 16). Statistical differences: * *p* < 0.05 compared to control (by post hoc Dunn’s multiple comparison test).

Concentrations of Ig types and IgG subtypes were measured in plasma samples of suckling rats on days 8 and 16 (Figure 2). On day 8, no significant differences were observed in total IgGs or IgM or IgA levels between the intervention groups (EcN-EVs and EVs-EcoR12) and the control group (Figure 2A). The interventions did not significantly modify IgG subtypes, nor the Th1/Th2 Ig ratio (IgG2b + IgG2c/IgG1 + IgG2a) (Figure 2A). In contrast, highly significant differences were observed on day 16 in animals given EcN or EcoR12 EVs (Figure 2B). Animals in the EcN EVs and EcoR12 EVs groups showed more elevated plasma IgG (*p* < 0.003), IgM (*p* < 0.001), and IgA (*p* < 0.001) concentrations compared to the control group.

**Figure 3:**
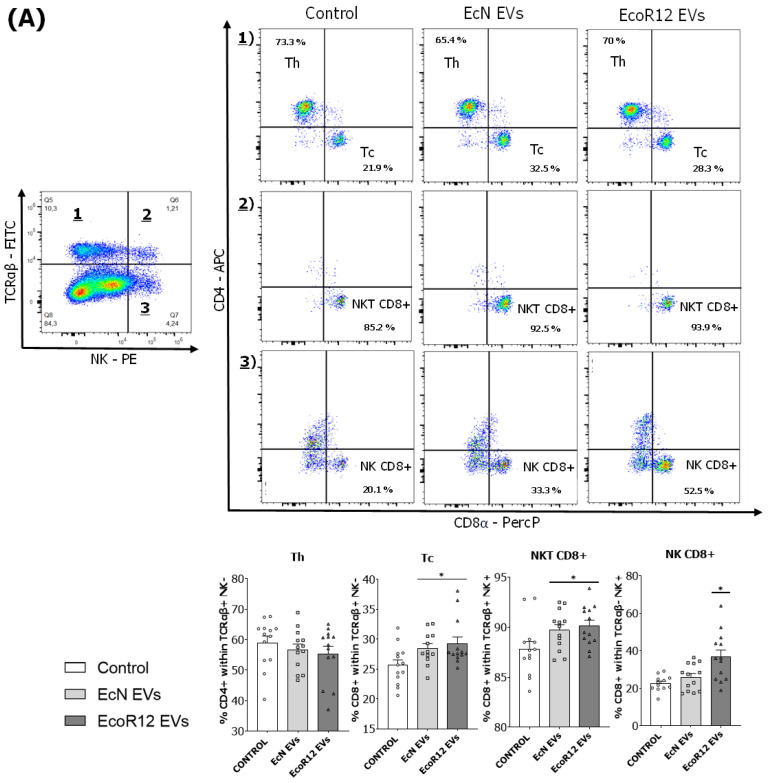

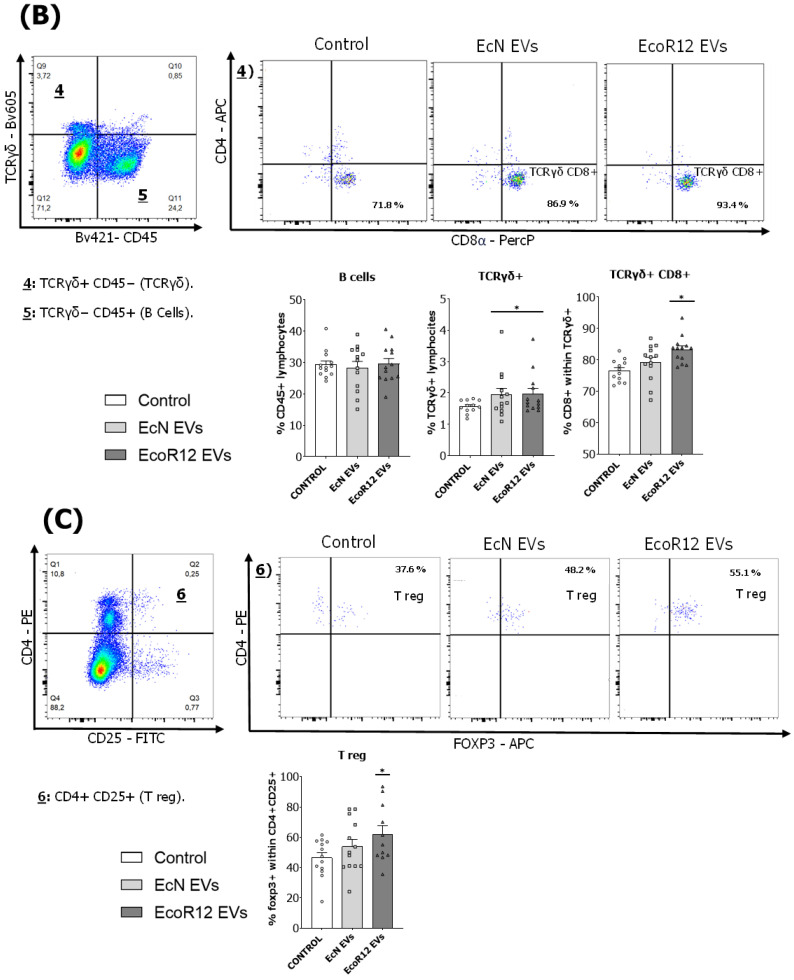
Effect of the interventions with EcN and EcoR12 EVs on the main spleen lymphocyte subsets. Flow cytometric analysis of lymphocytes isolated from spleen samples on the last day of the experimental period (day 16). The analyzed populations were: (**A**) Th cells (TCRαβ+ CD4+ NK−), Tc cells (TCRαβ+ CD8+ NK−), NKT cells (TCRαβ+ NK+, classified in CD8+ and CD8− subsets), and NK cells (TCRαβ− NK+, classified in CD8+ and CD8− subsets). (**B**) B cells (CD45RA+) and the T subset population expressing the receptor TCR-1 (TCRγδ+, classified in CD8+ and CD8− subsets). (**C**) Treg cells (CD4+ CD25+ FoxP3+). Abbreviations: Th, T-helper; Tc, T-cytotoxic; NK, natural killer; NKT, natural killer T; Treg, regulatory T cells. Representative dot plots for the three experimental groups (control, EcN EVs, and EcoR12 EVs) are shown for the analysis of each lymphocyte subtype gate. The percentage of positive cells is indicated in each quadrant or box. Results, presented in bar diagrams, are expressed as mean ± SEM (*n* = 12/group). Statistical differences: * *p* < 0.05 compared to the control (by post hoc Dunn’s multiple comparison test).

The increase in total IgG plasma levels on day 16 was due to an increase in all the IgG subtypes, without significant differences between the two interventions. Notably, both EcN and EcoR12 EVs raised plasma IgG2b levels by 7-fold (*p* < 0.002), IgG2c by 5-fold (*p* < 0.001), IgG1 by 4-fold (*p* < 0.002), and IgG2a levels by 2-fold (*p* < 0.001). Accordingly, both interventions promoted on day 16 a shift to the Th1 response as shown by the Th1/Th2 IgG ratio (Figure 3B).

### 3.3. Analysis of the Spleen Lymphoid Subsets

The relative percentages of splenic lymphocyte subsets were assessed on day 16 (Figure 3). No significant differences in the proportion of total B and T lymphocytes were apparent among the three experimental groups. However, the suckling rats administered daily with EcN EVs or EcoR12 EVs showed higher percentages of cytotoxic T cells (TCRαβ+ CD8+ NK−) (*p* < 0.05) and NKT cells (TCRαβ+ CD8+ NK+) (*p* < 0.05) in comparison to the control group (Figure 3A). Also, the percentage of NK cells (NK+ TCRαβ−) expressing CD8 was specifically elevated by the intervention with EcoR12 EVs (Figure 3A).

Interestingly, both interventions raised the proportion of the TCRγδ T cell subset (*p* < 0.05), principally the CD8+ subpopulation (*p* < 0.05 for the EcoR12 EVs group, while there was a tendency for the EcN EVs group) (Figure 3B). These results showed that the administration of EVs isolated from probiotic and commensal *E. coli* strains to neonatal rats stimulates the innate and adaptative immune systems by increasing the proportion of cytotoxic lymphocytes, which are important to fight against intracellular pathogens.

Moreover, both interventions (EcN EVs and EcoR12 EVs) tend to increase the proportion of Treg cells (CD4+, CD25+ FoxP3+) compared to the control group. Particularly, differences were statistically significant for the EcoR12 EVs group (Figure 3C).

### 3.4. Intestinal Gene Expression Analysis

**Figure 4:**
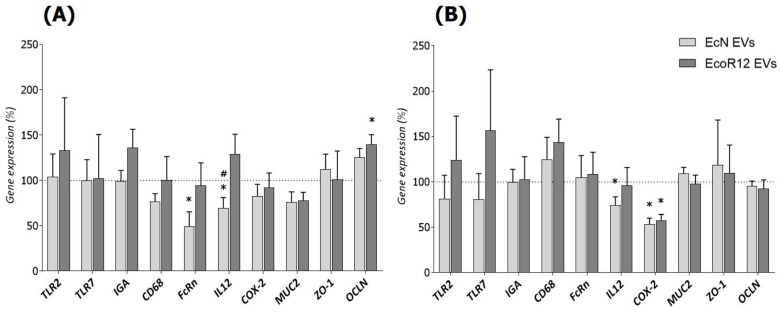
Modulation of gene expression by EcN and EcoR12 EVs in the small intestine of neonatal rats. The expression of genes related with intestinal function including markers of the immune system, maturation, inflammation, and barrier integrity was assessed by RT-qPCR at two points of the experimental period: (**A**) day 8 and (**B**) day 16. The relative expression in the interventional groups (EcN EVs, light gray bars; EcoR12 EVs, dark gray bars) was calculated with respect to the control group, which was assigned 100% transcription (indicated by a dotted line). Results are expressed as mean ± SEM (*n* = 12/group). Statistical differences: * *p* < 0.05 compared to control, # *p* < 0.05 between interventions (by post hoc Dunn’s multiple comparison test). Abbreviations: *TLR*, Toll-like receptor; *IGA*, immunoglobulin A; *CD68*, cluster of differentiation 68; *FcRn*, neonatal constant fragment receptor; *IL12*, interleukin-12; *COX-2*, cyclooxygenase-2; *MUC2*, mucin 2; *OCLN*, occludin; *ZO-1*, zonula occludens-1.

To further explore the impact of microbiota/probiotic EVs on the intestinal function, the expression levels of genes related to the immune system (*TLR2*, *TLR7*, *IGA*, *CD68*), intestinal maturation (*FcRn*), inflammation (*IL12*, *COX-2*), and gut barrier integrity/permeability (*MUC2*, *ZO-1*, and *OCLN*) were measured in the small intestine on days 8 and 16 (Figure 4).

Concerning immune-related genes, the administration of EcN or EcoR12 EVs did not significantly modify their expression. However, there was a tendency toward higher expression of *TLR2* and *TLR7* in the EcoR12 EVs group on day 16. Another gene related to the adaptative immunity that reflects the maturation state of the neonatal intestine is *FcRn*. The encoded protein acts as a receptor that allows the uptake of maternal IgG by the immature neonatal intestinal epithelium [11]. Notably, neonatal rats in the EcN EVs group showed lower *FcRn* expression levels (*p* < 0.05) on day 8 of life but levels comparable to the control group on day 16 of life. In line with the anti-inflammatory effects of the probiotic EcN, the administration of EcN EVs significantly reduced *IL12* (*p* < 0.05) expression at both times. Moreover, both interventions (EcN EVs and EcoR12 EVs) also significantly downregulated the proinflammatory enzyme COX-2 on day 16. Finally, considering the vesicle effects on genes involved in intestinal barrier integrity, both interventions increased occludin expression on day 8, with statistically significant differences observed for EcoR12 EVs (*p* < 0.05). On day 16, *OCLN* mRNA levels were comparable to those of the control group. At both intervention times, no significant differences in *ZO-1* or *MUC2* mRNA levels were observed between control and EV-treated groups.

### 3.5. Intestinal Histomorphometry

**Figure 5:**
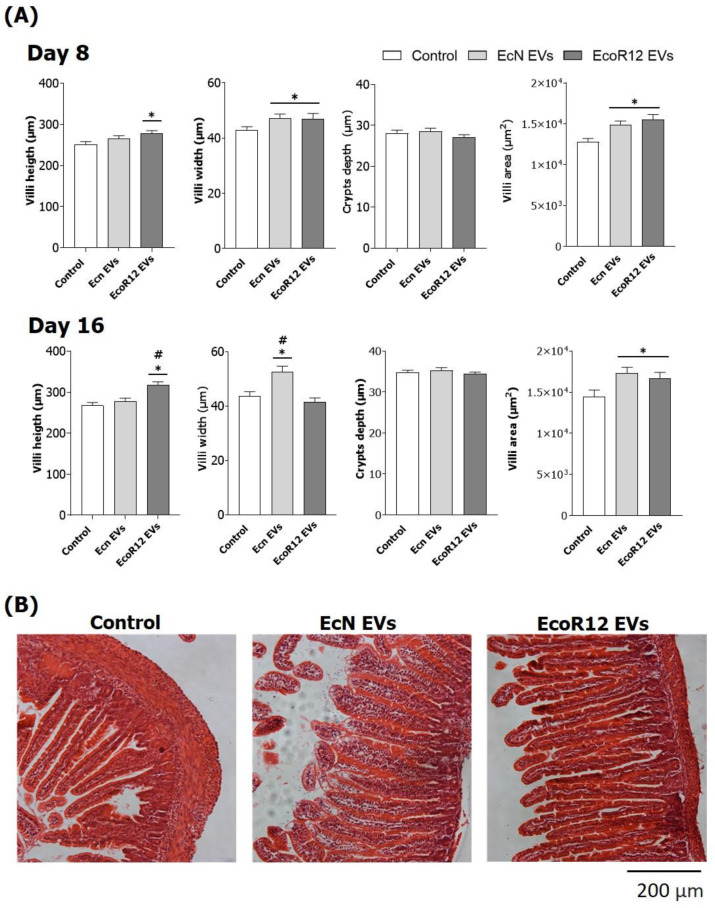
Effect of the interventions with EcN and EcoR12 EVs on intestinal histomorphometry variables on days 8 and 16. (**A**) Crypt depth and villi height, width, and area were measured with ImageJ v1.53t. At least 10 villi per animal were recorded. Results are expressed as mean ± SEM (*n* = 12/group). Statistical differences: * *p* < 0.05 compared to the control; # *p* < 0.05 between interventions (by post hoc Bonferroni test). (**B**) Representative images of distal jejunum sections of animals (day 16) stained with hematoxylin and eosin (10×) are shown.

The effects of microbiota EVs on the intestinal morphology of neonatal rats were assessed on days 8 and 16 (Figure 5).

Pups receiving daily EcN EVs displayed significantly greater villi width and villi area than the control (*p* < 0.05) at both time points. The intervention with EcoR12 EVs also promoted intestinal morphological changes. On day 8, animals receiving EcoR12 EVs showed significantly higher villi width, length, and area values compared to the control group (*p* < 0.05). As the newborn intestine developed (day 16), the increase in the villi height and area was sustained in EcoR12 EVs animals (*p* < 0.05). However, the villi width did not differ from that of the control group. None of the interventions influenced crypt depth.

## 4. Discussion

Neonate mammals are born with immature intestinal function and immunity. The immature small intestine has altered morphology and barrier properties. The high permeability of the neonatal intestine involves enhanced paracellular and transcellular passage of macromolecules. The transfer of microbial antigens contributes to the development and training of the newborn immature immune system, whereas the transfer of maternal milk IgA and IgGs provides immune protection during the neonatal period [10].

Studies using germ-free animals prove that colonization by gut microbiota plays a key role in intestinal maturation in neonates, affecting both the intestinal architecture and the immune system [13,39,40]. Although the diverse microbiota effects on the development and function of the intestinal barrier and immune system are well known, the molecular actors and mechanisms are not fully understood, particularly in the neonatal period. In the gut, interactions between the microbiota and host epithelial/immune cells are complex. Recent evidence indicates that interkingdom crosstalk in the gut primarily depends on multiple bioactive molecules and factors secreted by the gut microbiota [2,13]. In this context, EVs released by gut-resident microbes serve as vehicles for the secretion and delivery of bacterial effectors to host cells in a protected milieu provided by the vesicle membrane. Numerous studies in adult animals and cellular models of mature intestinal function have confirmed that microbiota EVs mediate the specific effects of gut-resident bacteria on host immune and defense responses [17]. Based on our previous results showing the immunomodulatory and barrier-strengthening activity of EVs from intestinal beneficial *E. coli* strains [33], we hypothesized that in the neonatal period microbiota EVs may contribute to the maturation and development of the newborn immune system.

It is known that at birth, the intestinal maturation stage differs among mammalian species. In particular, rodents are born with an immature gastrointestinal tract, and the development of the functional organ occurs after birth. For this reason, models based on neonatal mice or rats are appropriate for the study of maturation of intestinal barrier function and immunity [41,42].

The present study investigates the influence of interventions involving the oral administration of EVs derived from the probiotic EcN and the commensal EcoR12 on intestinal and immune maturation in the suckling rat model. Daily administration of EVs for two weeks (spanning from day 2 to day 16 of life) did not significantly affect the growth of animals compared to the control group. In general, the interventions did not cause substantial changes in the relative weight of organs (calculated as % of body weight). Nevertheless, the EcN EVs group showed a slight increase in spleen weight, especially on day 8, which might be attributed to enhanced immune activity. However, other factors such as greater irrigation cannot be ruled out. The EcoR12 EVs group displayed a slight enlargement of the empty cecum. Importantly, the stool features were not affected during the interventions, which indicates that administration of microbiota/probiotic EVs during the neonatal period was safe and well tolerated, as evidenced in experimental models with adult animals.

Microbiota EVs are known to influence mucosal and systemic immunity due to their content of numerous MAMPs that engage immune receptors expressed by host epithelial and immune cells of the innate and adaptative immune system [17,43]. Here, we assessed the effects of EVs isolated from beneficial gut resident *E. coli* strains on the humoral and cellular immune responses in neonatal rats. Oral administration of either EcN or EcoR12 EVs promoted a remarkably significant increase in the plasma levels of all Ig types (IgA, IgM, and IgG) after the 16-day intervention period compared to the control group. Overall, these results are in line with the well-known immunostimulatory and immunogenic properties of bacterial vesicles. In fact, bacterial EVs are being explored as native vaccines or vaccine adjuvants. Vaccines based on pathogen-derived EVs are as effective at raising antibody levels as the whole bacterial vaccine [44,45]. Moreover, the use of EVs from non-pathogenic bacterial strains as vaccine adjuvants in immunization processes has yielded promising results. In this context, vaccination with a mixture of bacterial EVs with antigens or EVs loaded with specific antigens triggers robust humoral and cellular immune responses after immunization, without adverse side effects [43,46]. Focusing on the IgG subtypes, our findings demonstrate that both interventions with microbiota EVs promoted a greater increase in Th1-type Igs (IgG2b and IgG2c) than in Th2-type IgGs (IgG1 and IgG2a). The immature immune system of the newborn is predominantly directed to a Th2 phenotype, and the immune responses shift towards a Th1 phenotype as the immune system develops [10,47]. Considering this, the increase in the Th1 / Th2 ratio induced by the interventions suggest that microbiota EVs may help immune system maturation in early life. This observation was supported by the changes induced by EcN and EcoR12 EVs in cellular immunity. Consistent with the increased production of antibodies and the switch to the Th1 phenotype, suckling rats receiving microbiota EVs displayed a higher proportion of splenic cytotoxic Tc, NK, and NKT cells than control animals. The impact of microbiota EVs also increased the proportion of a particular T cell subset expressing TRCϒδ, which was considered a primitive immune system that quickly mounts innate responses against pathogens and other injuries [48]. In addition, EVs from the commensal EcoR12 significantly increased the percentage of spleen Treg cells, a fact that is consistent with the previous results of our group, which showed the ability of EcoR12 EVs to activate dendritic cells to drive tolerogenic responses [26]. This Treg expansion in early life is of importance, as tolerance in this period is critical for proper immune development. Overall, the analysis of the spleen lymphoid subsets revealed that interventions with microbiota/probiotic EVs activate balanced immune responses that accelerate immune system maturation in early life, while preserving host tolerance against gut microbes.

It is known that in healthy mammalian adults, microbiota EVs cross the intestinal epithelial barrier and reach systemic circulation [16,49,50]. In the gut, migration of microbiota EVs across the intestinal epithelium is mediated by several pathways including transcellular and paracellular mechanisms. Passage of gut microbiota EVs through the paracellular route is favored under conditions of increased intestinal permeability [51,52]. Thus, the newborn immature intestine should enhance the access of EVs to the antigen-presenting cells of the lamina propria. The stimulation of the neonatal innate immune system by administered microbiota EVs could accelerate immune system development. This is consistent with the results shown in this study.

The results from the gene expression analysis of immune-related genes in intestinal tissue corroborated the immunomodulatory effects of microbiota EVs. Despite considerable variability between individuals within the same group, interventions with EcN EVs and particularly EcoR12 EVs tend to promote upregulation of the immune receptors TLR7 and TLR2, which recognize specific microbial molecules, single-stranded RNA, and bacterial peptidoglycan, respectively, and trigger effective immune responses. By this mechanism, microbiota EVs provide antigens that would contribute to the development and training of the neonatal immune system. However, although microbiota EVs help immune maturation, they do not cause intestinal inflammation, as shown by the mRNA levels of *CD68*, *IL12*, and *COX-2*. The glycoprotein CD68 is expressed by monocytes and macrophages and is normally used as a histochemical marker of inflammation associated with recruited macrophages [53]. Importantly, interventions with microbiota EVs did not increase *CD68* expression in the intestinal mucosa over control levels. Similarly, none of the interventions upregulated the proinflammatory cytokine IL-12. Moreover, EVs from the probiotic EcN specifically exerted a sustained anti-inflammatory effect by downregulating intestinal *IL12* mRNA levels. In this case, significant differences with respect to the control and EcoR12 EVs groups were observed from the first week of intervention and maintained until day 16. Furthermore, animals receiving EcN EVs also displayed reduced intestinal expression of *COX-2* during the intervention period. In the gut, this enzyme is induced in response to inflammatory stimuli and is involved in the production of prostaglandins that support the inflammatory process [54]. Although downregulation of *COX-2* by EcN EVs was already apparent on day 8, EVs from the commensal EcoR12 also reduced *COX-2* mRNA levels on day 16. Overall, these results agree with and support the anti-inflammatory activity of EcN EVs [33]. In addition, they show that interventions with EVs from beneficial gut microbiota potentiate humoral and cellular immunity in early life without causing noticeable inflammatory side effects.

Concerning the barrier properties of the rat immature intestine, one main feature is its high permeability, which allows transfer of Igs, antigens, and bioactive molecules via transcellular endocytosis and paracellular passage. Both routes provide a mechanism to prime immune system development [10]. Accordingly, the neonatal immature intestine displays lower expression of TJ proteins [9] and higher expression of FcRn [11] than the adult tissue. The maturation of the intestinal epithelial barrier in neonatal rodents has been associated with upregulation of TJ proteins and downregulation of the maternal IgG receptor. In this study, we show that interventions with microbiota EVs influence the expression of these markers towards a mature profile. In particular, the EcoR12 EVs group had significantly more *OCLN* expression than the control, and the EcN EVs group had significantly lower *FcRn* mRNA levels. Importantly, differences from the control group were only observed on day 8. On day 16, as the intestinal tract matured in the untreated control animals, the expression of these genes became similar in all groups. These results suggest that interventions with probiotic/microbiota EVs can accelerate intestinal maturation. In this context, the administration of microbiota EVs also seems to promote an intestinal trophic effect by increasing the villi area, primarily due to greater villi width (EcN EVs) or greater villi length (EcoR12 EVs). The positive impact of EV interventions on intestinal development would improve nutrient absorption in immature newborns.

The main advantage of interventions based on probiotic/microbiota EVs, in comparison to live probiotics, lies in the fact that they are non-replicative structures (free from bacteria), yet able to mediate the beneficial effects of the probiotics. This fact avoids the potential risk of bacterial translocation and infection in newborns or immunocompromised patients [55,56,57,58]. In this context, probiotic/microbiota EVs could represent a safe postbiotic strategy targeting intestinal immunity and maturation in early life. We are aware that the distinct maturation status of the human and rat neonatal intestine leads to a significant limitation of preclinical assays in suckling rats when transferring the derived results to humans. However, the neonatal rat model could better resemble the gut immaturity of human preterm newborns, a condition that negatively influences digestion, absorption of nutrients, and the pattern of gut microbiota. In turn, this condition contributes to disturbances in neural, functional, and immune development of the intestinal system, which predispose to intestinal complications and infections, such as necrotizing enterocolitis [57,58,59,60]. Overall, the findings presented here may suggest that interventions involving probiotic/microbiota EVs could be examined as a postbiotic strategy to prevent intestinal infections and help intestinal maturation to meet adequate nutrient absorption needs in preterm newborns. Also, such interventions could potentially be beneficial for enhancing the immune system and ameliorating allergies in infants or later in life. Clinical trials are needed to prove the benefits and safety of microbiota EV-based interventions in human infants.

## 5. Conclusions

In the present study, the intervention with probiotic/microbiota EVs in neonatal suckling rats has been shown to be a safe strategy to stimulate immunity and intestinal maturation in the neonatal period. Among the multiple benefits, EcN and EcoR12 EVs: (i) prime the newborn immune system at the cellular and humoral level to better fight against infections, (ii) preserve tolerance against the gut microbiota as depicted by their ability to increase the percentage of spleen Treg cells, (iii) elicit anti-inflammatory effects by reducing *IL12* and *COX-2* expression in the small intestine, (iv) promote intestinal maturation by modulating the expression of genes that affect transcytosis and the paracellular passage of macromolecules across the intestinal epithelium, and (v) promote trophic effects on the developing intestinal morphology by increasing the villi area. To our knowledge, this is the first preclinical assay evaluating the positive effects of microbiota/probiotic EVs on promoting immunity and intestinal maturation in early life.

## Figures and Tables

**Table 1:** Growth-associated measurements and relative weight of organs.

**Day 8**	**Control**	**EcN EVs**	**EcoR12 EVs**
Animal length (cm)	9.83 ± 0.15	9.64 ± 0.12	9.96 ± 0.07
Body (cm)	6.87 ± 0.11	6.86 ± 0.12	6.74 ± 0.11
Tail (cm)	3.05 ± 0.06	2.91 ± 0.05	3.15 ± 0.1
Body mass index (g/cm^2^)	0.24 ± 0.01	0.25 ± 0.01	0.27 ± 0.01 *
Lee index (g^0.33^/cm × 1000)	331.15 ± 2.96	331.23 ± 2.47	343.78 ± 4.41
Intestine weight (%)	3.19 ± 0.06	3.38 ± 0.11	3.16 ± 0.05
Intestine length (%)	231.57 ± 7.39	226.00 ± 6.17	212.31 ± 4.49
Cecum weight (%)	0.19 ± 0.04	0.19 ± 0.03	0.24 ± 0.04 *
Spleen weight (%)	0.48 ± 0.02	0.59 ± 0.03 *	0.50 ± 0.03
Liver weight (%)	2.85 ± 0.02	3.03 ± 0.05	2.86± 0.06
Thymus weight (%)	0.29± 0.01	0.29 ± 0.01	0.30 ± 0.01
Kidney weight (%)	0.71 ± 0.01	0.69 ± 0.01	0.70 ± 0.01
Heart weight (%)	0.69 ± 0.02	0.77 ± 0.02 *	0.72 ± 0.01
**Day 16**	**Control**	**EcN EVs**	**EcoR12 EVs**
Animal length (cm)	14.42 ± 0.22	14.36 ± 0.17	14.61 ± 0.04
Body (cm)	9.32 ± 0.12	9.32 ± 0.08	9.51 ± 0.06
Tail (cm)	5.1 ± 0.09	5.03 ± 0.09	5.1 ± 0.05
Body mass index (g/cm^2^)	0.33 ± 0.01	0.32 ± 0.01	0.31 ± 0.01
Lee index (g^0.33^/cm × 1000)	328.6 ± 3.15	326.98 ± 2.26	321.77 ± 2.9
Intestine weight (%)	2.94 ± 0.04	2.97 ± 0.04	2.95 ± 0.04
Intestine length (%)	126.08 ± 5.51	132.18 ± 3.67	129.27 ± 3.53
Cecum weight (%)	0.21 ± 0.01	0.24 ± 0.01	0.25 ± 0.01 *
Spleen weight (%)	0.44 ± 0.02	0.50 ± 0.01 *	0.41 ± 0.01
Liver weight (%)	3.08 ± 0.03	3.24 ± 0.1	3.20 ± 0.07
Thymus weight (%)	0.52 ± 0.01	0.47 ± 0.01	0.51 ± 0.01
Kidney weight (%)	0.61 ± 0.01	0.63 ± 0.02	0.60 ± 0.01
Heart weight (%)	0.62 ± 0.02	0.65 ± 0.02	0.62 ± 0.01

Data corresponding to relative organ weight are expressed as percentage of animal body weight. Results are expressed as mean ± SEM (*n* = 12 animals/group). * *p* < 0.05 compared to control (by post hoc Dunn’s multiple comparison test).

## Data Availability

The original contributions presented in the study are included in the article/Supplementary Materials. Further inquiries can be directed to the corresponding author.

## Supplementary Materials

The following supporting information can be downloaded at: https://www.mdpi.com/article/10.3390/nu15214701/s1, Table S1: Hematological variables.
