# Supplementary material for: Microbiota-Derived Extracellular Vesicles Promote Immunity and Intestinal Maturation in Suckling Rats
Source: Nutrients. 2023 Nov 6;15(21):4701. doi: 10.3390/nu15214701 (PMC10649425; doi:10.3390/nu15214701)
Supplement: Supplementary file 1 [file nutrients-15-04701-s001.zip › nutrients-2659196-supplementary.pdf]

Table S1. Hematological variables. The results are expressed as mean ± SEM (n= 12 animals/group). No statistical differences were observed between groups.

**Intervention time**	**Experimental groups**		
**Day 8**	**CON**	**EcN-EVs**	**EcoR12-EVs**
Leucocytes ($x10^9$/L)	2.84 ± 0.17	3.35 ± 0.35	2.75 ± 0.66
Lymphocytes (%)	64.12 ± 2.06	65.21 ± 2.16	64.69 ± 1.3
Monocytes (%)	6.69 ± 0.48	6.26 ± 0.38	6.63 ± 0.33
Granulocytes (5)	29.18 ± 1.83	28.53 ± 1.95	28.68 ± 1.26
Lymphocytes ($x10^9$/L)	1.78 ± 0.15	2.14 ± 0.24	1.71 ± 0.40
Monocytes ($x10^9$/L)	0.14 ± 0.01	0.13 ± 0.02	0.13 ± 0.05
Granulocytes ($x10^9$/L)	0.92 ± 0.05	1.08 ± 0.12	0.91 ± 0.21
Erythrocytes ($x10^{12}$/L)	3.10 ± 0.08	2.87 ± 0.09	2.90 ± 0.14
Haemoglobin (g/L)	8.75 ± 0.20	8.65 ± 0.25	8.38 ± 0.41
Haematocrit (%)	30.08 ± 0.76	29.25 ± 0.79	28.27 ± 1.43
MCV (fL)	97.81 ± 1.06	102.32 ± 1.56	97.81 ± 1.01
MCH (pg)	28.30 ± 0.53	30.21 ± 0.78	28.93 ± 0.54
Platelets ($x10^9$/L)	400.85 ± 43.06	276.38 ± 46.82	311.92 ± 65.39
**Day 16**			
Leucocytes ($x10^9$/L)	1.78 ± 0.12	2.05 ± 0.20	2.46 ± 0.29
Lymphocytes (%)	67.93 ± 1.01	66.81 ± 1.16	67.22 ± 1.54
Monocytes (%)	5.66 ± 0.25	5.95 ± 0.33	5.73 ± 0.42
Granulocytes (5)	26.42 ± 0.94	27.15 ± 1.19	27.05 ± 1.26
Lymphocytes ($x10^9$/L)	1.15 ± 0.09	1.32 ± 0.13	1.58 ± 0.19
Monocytes ($x10^9$/L)	0.05 ± 0.02	0.07 ± 0.02	0.09 ± 0.03
Granulocytes ($x10^9$/L)	0.58 ± 0.04	0.66 ± 0.06	0.78 ± 0.09
Erythrocytes ($x10^{12}$/L)	4.17 ± 0.09	3.71 ± 0.37	4.14 ± 0.06
Haemoglobin (g/L)	8.82 ± 0.24	7.65 ± 0.67	8.41 ± 0.22
Haematocrit (%)	28.29 ± 0.71	25.11 ± 2.47	27.48 ± 0.40
MCV (fL)	67.91 ± 0.73	68.47 ± 0.75	66.42 ± 0.47
MCH (pg)	20.89 ± 0.51	22.70 ± 2.46	20.24 ± 0.53
Platelets ($x10^9$/L)	503.73 ± 50.17	645. 70 ± 100.75	602.83 ± 53.93
